# Distinct Mammalian Precursors Are Committed to Generate Neurons with Defined Dendritic Projection Patterns 

**DOI:** 10.1371/journal.pbio.0050300

**Published:** 2007-11-13

**Authors:** Wolfgang Kelsch, Colleen P Mosley, Chia-Wei Lin, Carlos Lois

**Affiliations:** 1 Picower Institute for Learning and Memory, Massachusetts Institute of Technology, Cambridge, Massachusetts, United States of America; 2 Department of Brain and Cognitive Sciences, Massachusetts Institute of Technology, Cambridge, Massachusetts, United States of America; 3 Department of Biology, Massachusetts Institute of Technology, Cambridge, Massachusetts, United States of America; Massachusetts General Hospital, United States of America

## Abstract

The mechanisms that regulate how dendrites target different neurons to establish connections with specific cell types remain largely unknown. In particular, the formation of cell-type–specific connectivity during postnatal neurogenesis could be either determined by the local environment of the mature neuronal circuit or by cell-autonomous properties of the immature neurons, already determined by their precursors. Using retroviral fate mapping, we studied the lamina-specific dendritic targeting of one neuronal type as defined by its morphology and intrinsic somatic electrical properties in neonatal and adult neurogenesis. Fate mapping revealed the existence of two separate populations of neuronal precursors that gave rise to the same neuronal type with two distinct patterns of dendritic targeting—innervating either a deep or superficial lamina, where they connect to different types of principal neurons. Furthermore, heterochronic and heterotopic transplantation demonstrated that these precursors were largely restricted to generate neurons with a predetermined pattern of dendritic targeting that was independent of the host environment. Our results demonstrate that, at least in the neonatal and adult mammalian brain, the pattern of dendritic targeting of a given neuron is a cell-autonomous property of their precursors.

## Author Summary

The mammalian brain contains a large number of different classes of neurons that are connected in a specific manner. A long-standing question is how such stereotyped connections emerge during the assembly of the brain. Here, we investigated whether neonatal and adult brain stem cells give rise to neurons whose connections can be influenced by the partners that they encounter while maturing, or alternatively, whether these connections are predetermined from the moment that a neuron is born. We observed the existence of distinct populations of precursor cells committed to generating neurons with a specific pattern of connections. Furthermore, the pattern of connections formed by these neurons was largely independent of the environment in which the neurons matured. These results have important implications for the formation of neuronal circuits, as they indicate that the connections of a new neuron can be determined in their precursors. In particular, these observations suggest that for neuronal replacement therapies to be successful, it will be necessary to understand the genetic programs that control how stem cells are prespecified to produce neurons with a stereotypic pattern of connections.

## Abstract 3

How do adult-born neurons connect into specific brain circuits? The authors show that distinct progenitors are committed to generate the same neuron type, but with different prespecified projection patterns.

## Introduction

Dendrites are the major source of synaptic input for neurons. Thus, the specific computation that a neuron can accomplish is largely determined by the synaptic partners that contact its dendrites. In many regions of the central nervous system, including the cortex, spinal cord, retina, and olfactory bulb [1–4], neurons that share a common morphology and similar microenvironment have dendrites that target synaptic partners in different laminae. Although significant advances have been made in understanding the mechanisms that control axonal pathfinding during development [5,6], relatively less is known about the regulation of dendritic connectivity [7–9]. In recent years, some of the cellular mechanisms involved in dendritic growth into specific laminae have been characterized [4,7,9–11]. In addition, some cell-adhesion molecules involved in the formation of cell type–specific dendritic connectivity have been identified [8]. However, it remains unknown whether lamina-specific targeting is a cell-autonomous property of immature neurons, or alternatively, determined by the cellular environment in which the neurons differentiate. How lamina-specific dendritic targeting is specified is a particularly interesting question for neurons generated in the neonatal or adult period, because these new neurons have to integrate into a functioning, mature neuronal circuit.

**Figure 1:**
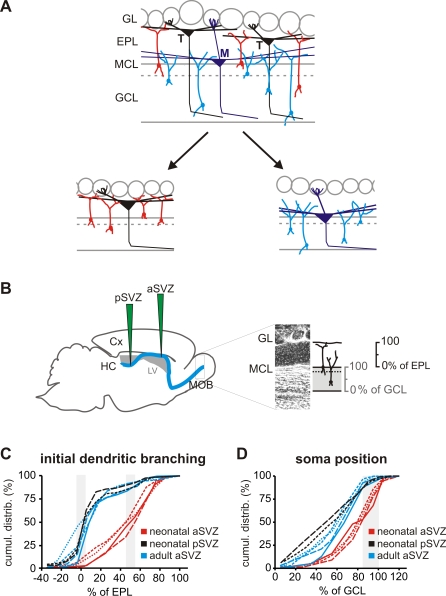
Distinct Precursors Gave Rise to Separate Populations of GCs with Initial Branching of the Apical Dendrite in Different Laminae of the EPL (A) GCs extend apical dendrites towards the EPL where they form synapses with the dendrites of mitral (M) and tufted (T) cells. The dendrites of one subpopulation of GCs (blue) initially branch near the MCL, whereas the dendrites of the other population of GCs (red) first branch in the superficial lamina of the EPL. Mitral cells (M) extend their lateral dendrites in the deep lamina of the EPL, whereas (middle) tufted cells (T) soma are located in the superficial lamina of the EPL, and extend their dendrites in the superficial EPL. GL, glomerular layer. (B) Sites for labeling of GC precursors in the aSVZ and pSVZ with a retroviral vector expressing GFP. For quantification of the position of the soma and of the initial branching of the apical dendrite, the GCL and the EPL were divided in percentages, with 0% being the deep and 100% being the superficial border. (C) Cumulative distribution of initial branching points of the GC apical dendrite. A total of 50% of GCs originating in the anterior aSVZ of neonatal animals initially branched after passing 50% through the EPL. In contrast, over half the GCs derived from the pSVZ of neonatal animals, and GCs derived from the adult aSVZ, branched before they have projected through 10% of the EPL. Different time points were analyzed for aSVZ at 14 (dotted line [^… .^]), 28 (short-dashed line [- - -]), 56 (long-dashed line [^_ _ _^]), and 112 d.p.i. (solid line [^___^]), and for pSVZ at 35 (short-dashed line [- - -]) and 63 d.p.i. (long-dashed line [^_ _ _^]). (D) Cumulative distribution of the soma position in the GCL of GCs generated from the different SVZ precursor populations shown in (C). Same labeling as in (C).

In this study, we examined the regulation of differential dendritic targeting of one neuronal type, the granule cell (GC) neuron of the olfactory bulb. GCs are axonless inhibitory interneurons that are continuously incorporated into the olfactory bulb throughout life [12,13]. GCs form dendro-dendritic synapses with the two types of projection neurons of the bulb, the mitral and the tufted cells (Figure 1A). GCs have distinct patterns of dendritic targeting—innervating either a deep or superficial lamina, where they connect to either mitral or tufted cells, respectively [14–16].

How is the lamina-specific dendritic targeting regulated in GCs? Throughout postnatal life, GCs are generated from neuronal precursors that proliferate in the subventricular zone (SVZ) and give rise to neuroblasts that migrate through the rostral migratory stream (RMS) into the olfactory bulb [12,13]. One possible scenario is that local cues within the olfactory bulb regulate the lamina-specific dendritic targeting of immature GCs at the time of their differentiation. Another possibility is that immature GCs are already committed to specific patterns of dendritic arborization at the moment of their birth in the SVZ, before they reach the bulb. To investigate these possibilities, we performed retroviral fate-mapping and transplantation experiments to test whether different populations of precursors give rise to GCs with lamina-specific dendritic arborizations. We discovered that the SVZ contained distinct populations of neuronal precursors committed to generate GCs with dendritic targeting to specific laminae. Furthermore, these precursors were largely restricted in their developmental potential with respect to dendritic targeting even when challenged with a SVZ microenvironment that normally generated GCs with dendrites that targeted the other lamina. Our results demonstrate that, in the neonatal and adult mammalian brain, the pattern of dendritic targeting of a given neuron can be a cell-intrinsic property that is already determined at the time of its birth. These findings have important implications both for assembly of neuronal circuits, and for the potential uses of adult neuronal stem cells in cell replacement therapies.

## Results

### Distinct Populations of Neonatal SVZ Precursors Gave Rise to GCs with Lamina-Specific Dendritic Targeting

In the neonatal brain, precursors in the SVZ give rise to GCs that integrate into the olfactory bulb. Most GCs have an apical dendrite that branches either in the deep or the superficial lamina of the external plexiform layer (EPL) (Figure 1A). To investigate whether precursors along the entire length of the neonatal SVZ have a similar developmental potential to give rise to GCs with a specific pattern of dendritic targeting, we used retroviral fate mapping to label precursors located in either one region of the anterior or posterior SVZ (aSVZ and pSVZ, respectively) of neonatal rats (Figures 1B and S1D). Oncoretroviruses have a half-life of only 6 h [17] and infect only actively dividing cells. Since the transient amplifying cell population is the most abundant actively dividing cell type in the SVZ, and the direct precursor to immature GCs, oncoretroviral infection is very effective for birth dating a single cohort of immature neurons [18]. Indeed, after infecting SVZ precursors with oncoretroviral vectors, we detected a single wave of labeled precursors that reached the olfactory bulb together. At 14 and 21 days postinfection (d.p.i.), only 4.8% (*n* = 1,218) and 1.3% (*n* = 780), respectively, of the total number of retrovirally labeled cells were still found in the RMS of the olfactory bulb.

**Figure 2:**
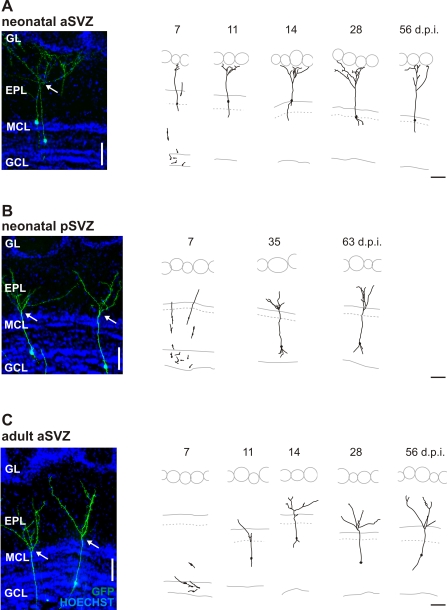
GC Reconstructions Revealed Lamina-Specific Dendritic Targeting (A) Different types of GCs were generated from distinct precursors in the SVZ labeled with a GFP (green) expressing retrovirus. Hoechst 33258 (blue) revealed the anatomical layers. Precursors infected in the aSVZ of neonatal rats gave rise to GCs whose first dendritic branching point (arrow) was in the superficial EPL. (B) Infection of precursors in the pSVZ of neonatal rats generated GCs whose first dendritic branching point was the deep EPL (arrow). (C) Precursors that were infected in the aSVZ of adult rats generated GCs with apical dendrites that branched in the deep layers of the EPL (arrow). The morphological characteristics of newly generated GCs were established soon after their differentiation and maintained throughout maturity. Time course of GC maturation in the olfactory bulb for precursors from neonatal aSVZ (A), neonatal pSVZ (B), and adult aSVZ (C) at different time points (in d.p.i.) is shown. All bars indicate 100 μm.

An oncoretroviral vector expressing green fluorescent protein (GFP) was stereotactically injected into the aSVZ or the pSVZ, and the morphology of GFP-positive (GFP^+^) GCs in the olfactory bulb was assessed 28 d.p.i., when they had acquired a mature neuronal morphology (Figure 2). We observed that the apical dendrite of GCs generated in the aSVZ of neonatal animals branched predominantly in the superficial lamina of the EPL, whereas the branches of the apical dendrite of GCs generated in the pSVZ of neonatal animals were mostly confined to the deep lamina of the EPL (Figure 2). This result was found to be independent of the strain or sex of the animals (see Materials and Methods and Figure S1A). Furthermore, we confirmed that the lamina-specific targeting and position of the initial dendritic branch point of new GCs was observed at all stages of their maturation (Figures 1C and 2).

To quantify these findings, we measured the position of the initial branch point of the apical dendrite of GFP^+^ GCs from each precursor population (*n* = 250 for each time point after injection and each site of injection). To determine the position of the initial dendritic branch point, the width of the EPL was assigned percentages, with 0% being the mitral cell layer (MCL), and 100% being the border between the EPL and glomerular layer (GL) (Figure 1B). The EPL was then divided into 10% steps, and the position of the initial branch point of the apical dendrite was assessed using this scale. The cumulative distribution of the initial branch-point positions revealed that the dendrites of GCs generated from neonatal aSVZ precursors branched superficially, with a median initial branch point approximately halfway through the EPL (50.1% of EPL, median for all time points, *n* = 1,000; Figure 1C). In contrast, neonatal pSVZ precursors gave rise to GCs with apical dendrites that branched in the deep EPL, with a median branch point close to the MCL (2% of EPL, median for all time points, *n* = 500; Figure 1C). The distribution of the initial dendritic branching point was significantly different for neonatal-generated GCs from the aSVZ and pSVZ (*p* < 0.0001; *n* = 1,000 and 500, respectively). It is important to note that retroviral injection into the aSVZ is also likely to label some of the precursors that originate in other parts of the SVZ (e.g., pSVZ), but that proliferate in the aSVZ while they migrate through it on their way towards the olfactory bulb. However, after injecting into the aSVZ, the number of transit-proliferating cells that originated from the pSVZ was less than 5% (*n* = 500). As shown in Figure 1C, the cumulative distribution of the initial branching point for the pSVZ cells was very steep close to the MCL, whereas that of the aSVZ cells was nearly flat in this same region. In summary, fate-mapping experiments demonstrate the existence of at least two distinct precursor populations in the neonatal SVZ, each committed to generate GCs with specific patterns of dendritic targeting in the olfactory bulb.

### Adult SVZ Precursors Continue to Generate GCs with Lamina-Specific Dendritic Targeting

Because new GCs continue to be added into the olfactory bulb throughout life, we also investigated whether distinct GC precursor populations persisted into adulthood. Similar to our previous experiments, we observed that oncoretroviral infection of the adult aSVZ led to the efficient labeling of a single cohort of immature GCs in the olfactory bulb. At 14 and 21 d after retroviral labeling of precursors in adult rats, only 8.7% (*n* = 922) and 0.8% (*n* = 615), respectively, of the total number of cells labeled were still found migrating in the RMS of the olfactory bulb.

Retroviral fate mapping revealed that GCs generated from precursors located in the aSVZ of adult animals had apical dendrites that branched predominantly in the deep lamina of the EPL (Figure 2). Quantification of this result indicated that the dendrites of GCs born from adult aSVZ precursors had a median branch-point position close to the MCL (4.5% of EPL, median for all time points, *n* = 1,000; Figure 1C), similar to the dendritic branching pattern of GCs born from neonatal pSVZ precursors. The distribution of the initial dendritic branching point was significantly different for neonatal- and adult-generated GCs from the aSVZ (*p* < 0.0001; *n* = 1,000, respectively). Again, this result was found to be independent of the strain or sex of the animals (see Materials and Methods and Figure S1A). Furthermore, as discussed for the neonatal animals, we confirmed that the lamina-specific targeting and position of the initial dendritic branch point of new GCs was observed at different stages of their maturation (Figures 1C and 2C). Interestingly, the normalized soma position of GCs from adult aSVZ was between that of GCs from neonatal aSVZ and pSVZ (Figure 1D) when compared at the same age (postnatal day [P]69–70).

We also investigated the fate of actively dividing precursors in the pSVZ and in the sector of the RMS rostral to the SVZ in adult animals. The dividing precursors in these two regions both gave rise mainly to GCs with deep dendritic targeting even though both regions also contained some GCs with superficial dendritic targeting (see Figure S1B). This observation suggested that superficially branching GCs are still generated in the adult. However, using the retroviral labeling technique described in this study, we could not detect a SVZ region in the adult animal that exclusively contained actively dividing precursors committed to the generation of superficially branching GCs. Two recent studies [19,20] observed that generation of superficial GCs peaks in the neonatal period and decreases thereafter, consistent with our findings that precursors labeled in the different regions of the adult RMS and SVZ mainly gave rise to deep-targeting GCs. Taken together, these findings indicate that distinct populations of precursors in the SVZ gave rise to GCs that target either the deep or superficial EPL.

### Dendritic Morphology and Synaptic Contacts of GCs Derived from Different SVZ Precursor Populations

**Figure 3:**
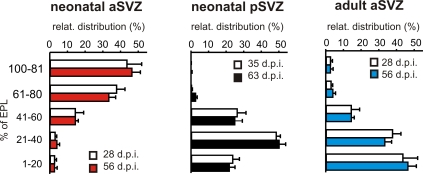
GC Spines in the EPL Were Confined to a Specific Lamina The relative distribution of spines for GCs generated in newborn animals (that were reconstructed in Figure 2.) is plotted at different time points (in d.p.i.). Precursors in neonatal animals from the aSVZ gave rise to GCs with spines confined to the superficial lamina of the EPL, whereas those from the neonatal pSVZ and adult aSVZ gave rise to GCs with spines in the deep lamina.

In our initial experiments, we quantified the position of the initial branch point of the apical dendrite as a surrogate measure of lamina-specific dendritic targeting. To obtain a more comprehensive view of the dendritic targeting of new GCs generated from different SVZ precursor populations, we reconstructed the dendritic arbors of retrovirally labeled GFP^+^ GCs, selecting cells that displayed complete dendrites without apparent truncation due to tissue sectioning (Figure 2; *n* = 10 GCs for each time point and condition). Most GCs generated from neonatal pSVZ and adult aSVZ precursors had fine dendritic branches that ended in the deep lamina of the EPL, whereas GCs generated from neonatal aSVZ had fine dendritic branches that were located in the superficial EPL (Figure 2). GC reconstructions indicated that their dendritic arbors were largely confined to specific laminae, thereby suggesting that these GCs establish synaptic contacts in specific laminae. An alternative possibility is that GC synapses are not uniformly distributed throughout the dendritic arbor, and that the lamina-specific elaboration of the terminal fine dendritic branches did not reflect lamina-specific innervation. To further investigate these possibilities, we quantified the distribution of spine protrusions studding the branches of the apical dendritic arbor of GCs in the EPL using single-cell GC reconstructions (*n* = 10 GCs for each time point and condition). Dendritic spines are the major sites of excitatory synaptic transmission in the mammalian brain [21] and are thought to be morphological correlates of synapses. In GCs of the olfactory bulb, dendritic spines are the primary sites of both the inputs and outputs of dendro-dendritic synapses to and from mitral and tufted cells [16]. Few spines were found along the primary apical dendrite prior to the initial dendritic branching point. We found that neonatal pSVZ and adult aSVZ precursors generated GCs with spine protrusions confined to the deep lamina of the EPL (Figure 3), consistent with their pattern of dendritic arborization. Furthermore, neonatal aSVZ generated GCs with spine protrusions confined to the superficial lamina of the EPL (Figure 3).

To further investigate the distribution of synaptic contacts in GC dendritic arbors, we also labeled the postsynaptic sites of glutamatergic synaptic inputs into GCs by expressing a genetic marker, PSD-95 fused to GFP, in GCs using an oncoretroviral vector. PSD-95 is a major scaffolding component of the postsynaptic density at excitatory synapses [22]. When GFP-tagged PSD95 is expressed in neurons, it clusters at the postsynaptic densities of glutamatergic synapses [23–25]. We found that the distribution of postsynaptic sites in GCs generated from precursors in the neonatal aSVZ and adult aSVZ, as labeled using PSD-95:GFP, was very similar to that described above for spine protrusions (see Figure S2 and Text S1). Thus, two independent methods indicate that distinct neuronal precursors in the postnatal SVZ generate GCs with lamina-specific patterns of synaptic innervation.

### Deep and Superficial GCs Share Intrinsic Somatic Electrical Properties

**Figure 4:**
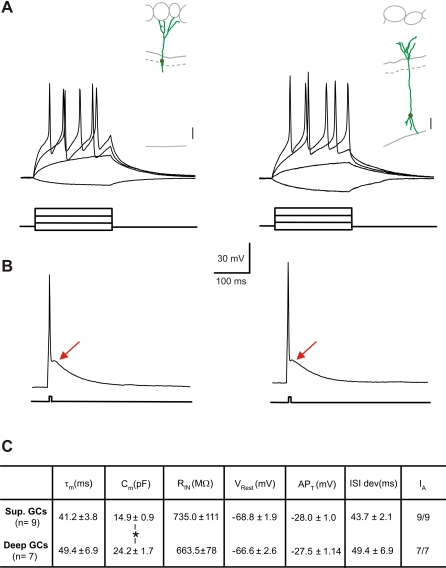
Deep and Superficial Dendritic Targeting GCs Intrinsic Somatic Electrical Properties (A) Targeted whole-cell recordings of GFP^+^ GCs (labeled either in the neonatal aSVZ or pSVZ) were performed (21–23 d.p.i.) and reconstructed after recordings. Both superficial (left panel) and deep (right panel) targeting GCs had a delayed firing pattern (200-ms current injection in 0.1-nA increments). (B) Both superficial (left panel) and deep (right panel) targeting GCs displayed an afterdepolarization current following a single spike (5-ms current injection in 0.4-nA increments). (C) The table summarizes passive and active membrane properties determined from superficial and deep targeting GCs. All determined parameters except membrane capacitance were similar for GCs with either deep or superficial dendritic targeting. τ_m_ is the membrane constant in milliseconds, *C*_m_ the membrane capacitance in picofarads, *R*_IN_ the input resistance in megaohms, *V*_rest_ the resting membrane potential in millivolts, AP_T_ the action potential threshold in millivolts, ISI, the interspike interval in millivolts, and *I*_A_ is the *I*_A_ current.

GCs with deep and superficial branching dendrites labeled by retroviral infections shared similar cell morphology (Figure 2). We explored whether GCs with deep or superficial dendritic targeting may also share intrinsic somatic electrical properties that provide a useful criterion towards neuronal type classification [26–28]. Towards this aim, we performed targeted whole-cell recordings in acute slice preparations from GFP^+^ neurons with either superficial or deep dendritic targeting (21–23 d.p.i.) that were labeled in the aSVZ or pSVZ, respectively, in neonatal animals (Figure 4). Both deep and superficial neurons had similar delayed firing patterns (Figure 4A) and afterdepolarizations (Figure 4B). In addition, cells with either deep or superficial dendrites had *I*_A_ currents that were abolished by exposure to 10 mM of 4-aminopyridine (unpublished data) and similar membrane properties (Figure 4C). The membrane capacitance of deep branching cells was larger than that of the superficial cells, most likely due to their differences in membrane surface. In summary, these data confirm that the new neurons with deep and superficial dendritic targeting not only shared a common morphology, but both also had similar intrinsic somatic electrical properties typical for GCs [29–32]. Thus, these observations suggest that distinct precursors in the SVZ are committed to giving rise to a single neuronal type with two alternative patterns of dendritic targeting.

### Fate Determination of GC Precursors

**Figure 5:**
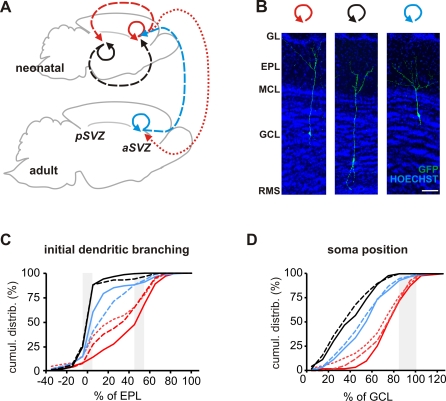
Transplantations of Distinct SVZ Regions Revealed Determination of Initial Dendritic Branching Patterns of GCs in Their Precursors (A) The diagram shows the different transplantation conditions from aSVZ and pSVZ donor tissue of neonatal and adult animals. (B) GCs detected 35 d post-transplantation after isochronic, isotopic transplantation of aSVZ of neonatal GFP^+^ rats to neonatal host aSVZ (left), pSVZ of neonatal GFP^+^ rats to neonatal host pSVZ (middle), and aSVZ of adult GFP^+^ rats to adult host aSVZ (right). The bar indicates 100 μm. (C) Cumulative distribution of initial branching point of the apical dendrite in the EPL of GCs generated from transplanted SVZ regions as described in (A) (same labeling as in [A] for the different transplantation conditions). (D) Cumulative distribution of the soma position in the GCL of GCs generated from transplantations shown in (C).

The factors regulating the lamina-specific targeting of GCs in the olfactory bulb are currently unknown. Our results raised the possibility that different populations of SVZ precursors may be committed to generate GCs with a particular pattern of dendritic targeting. Alternatively, local cues in the SVZ or olfactory bulb may control the developmental program of GC precursors or migrating GCs with respect to dendritic targeting. To investigate these possibilities, we performed heterochronic and heterotopic transplantations of different SVZ regions to examine whether their progeny adopt a different fate when challenged with new environments. In transplantation experiments, we isolated explants from three different sources, the neonatal aSVZ or pSVZ and adult aSVZ of GFP^+^ transgenic donor rats [33], and stereotactically injected them into the neonatal aSVZ or pSVZ and adult aSVZ of wild-type host rats (Figure 5A). The dendritic targeting of GFP^+^ GCs in the olfactory bulb was assessed 35 d post-transplantation to allow for their full maturation (Figure 5) (*n* = 200–731 neurons from 6–16 host hemispheres, per experiment).

In order to validate that precursors in the SVZ retain their endogenous ability to generate GCs with lamina-specific dendritic targeting, we first performed isochronic, isotopic transplantation experiments in which the aSVZ from neonatal donors was grafted into the aSVZ of neonatal hosts. GCs derived from these transplanted precursors extended dendrites that targeted the superficial lamina of the EPL (Figure 5B and 5C), confirming the results of our retroviral fate-mapping experiments (Figure 1D and 1E). Similarly, isochronic, isotopic transplantation of either pSVZ of neonatal donors into the pSVZ of neonatal hosts or of the aSVZ of adult donors into the aSVZ of adult hosts resulted in GFP^+^ GCs whose dendrites targeted the deep lamina of the EPL (Figure 5B and 5C). These data demonstrate that the transplantation procedure itself did not perturb the endogenous developmental potential of GC precursors with respect to lamina-specific dendritic targeting.

We then tested whether heterochronic and heterotopic transplantation of distinct SVZ regions can give rise to GCs with different dendritic targeting when exposed to a different environment. After heterochronic transplantation of precursors from adult aSVZ donors to neonatal aSVZ hosts as well as heterotopic transplantation from neonatal pSVZ to neonatal aSVZ, GFP^+^ GCs largely maintained their deep initial branching point (Figure 5C). After transplantation of precursors from neonatal aSVZ to adult aSVZ or to neonatal pSVZ, the GCs largely maintained their fate and had a superficial initial dendritic branching point (Figure 5C). We only observed a small increase in the number of GFP^+^ GCs that had a deeper initial dendritic branching point compared to isochronic and isotopic transplantation from the neonatal aSVZ donors.

To quantify these observations, we counted the number of neurons whose initial branching point occurred below or above the midpoint of the EPL. We then calculated the ratio of cells that branched below the EPL midpoint threshold to the total number of GFP^+^ GCs. We measured this ratio for GCs derived from the same donor grafted heterochronically or isochronically, and then calculated the change in the ratio of GCs that initially branched below the EPL midpoint, and expressed it as a percentage.

When neonatal aSVZ was transplanted into adult aSVZ, we observed a small increase (17.1%, Mann-Whitney test: *p* < 0.05%) of GCs that branched below the EPL midpoint, when compared to isochronic transplantation (neonatal aSVZ to neonatal aSVZ) from the same donor. Heterotopic transplantation from neonatal aSVZ to neonatal pSVZ resulted in 15.8% change (*p* = 0.13; not statistically significant) of GCs that branched below the EPL midpoint (Figure 5C). When we transplanted neonatal pSVZ into neonatal aSVZ, we observed a very small change (4%, not statistically significant) of GCs that branched above the EPL midpoint (Figure 5C). The small change observed in the population of GC precursors from the neonatal aSVZ after transplantation could be due to some partial phenotypic plasticity of these neonatal progenitors, or alternatively, to the transplantation procedure used in these experiments (see Discussion). Finally, heterochronic transplantation of adult aSVZ into neonatal aSVZ host did not induce a change (0.4%, not statistically significant) in GCs that branched below the EPL midpoint, when compared to isochronic transplantation (adult aSVZ to adult aSVZ) from the same donor (Figure 5C). In summary, even though some small changes can occur when the precursors are challenged with a new environment, the vast majority of new GCs derived from the different SVZ regions maintained their pattern of dendritic targeting.

**Figure 6:**
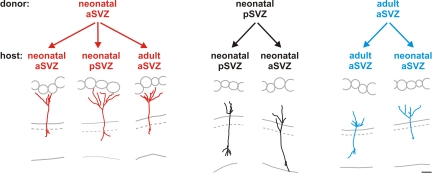
Reconstructions of Transplanted GCs Revealed That They Maintained Their Lamina-Specific Dendritic Targeting after Transplantation GC reconstructions revealed that the new GCs maintained their lamina-specific dendritic targeting after the different transplantation conditions indicated. The bar indicates 100 μm.

**Figure 7:**
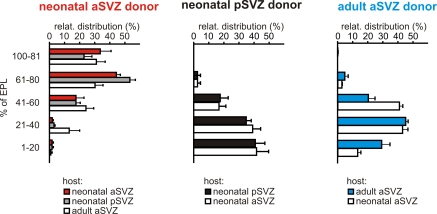
Lamina-Specific Distribution of GC Spines in the EPL Is Maintained after Transplantation Relative distribution of GC spines in the EPL from aSVZ or pSVZ of neonatal, and aSVZ of adult donors (for the neurons reconstructed in Figure 6.).

In order to obtain a more complete picture of the lamina-specific targeting of the apical dendritic arbor of transplant-derived GCs, we reconstructed the morphology of representative GFP^+^ GCs, as described above (Figure 6, *n* = 10 cells per condition). Neuronal reconstructions revealed that precursors that generate GCs with deep dendritic targeting in their native environment still gave rise to GCs with targeting of the deep lamina after heterochronic or heterotopic transplantation (Figure 6). The same observation applied to precursors that generate GCs with superficial dendritic targeting when challenged with a different proliferative environment (Figure 6). Finally, to confirm that the lamina-specific dendritic targeting reflects a lamina-specific distribution of synapses in these GCs, we determined the distribution of spines within the dendritic arbors of transplant-derived GCs. Similar to our previous findings, the distribution of dendritic spines reflected the lamina-specific dendritic targeting of transplanted GCs (Figure 7). Thus, our transplantation experiments indicate that precursors in the neonatal and adult animals appeared to be committed to generate GCs with a specific pattern of dendrite branching that was not modified by exposure to a brain environment that normally generated GCs with opposite dendritic targeting.

## Discussion

In this study, we investigated lamina-specific dendritic targeting of neurons generated in neonatal and adult rats. In particular, we tested whether local factors determine the dendritic targeting or, alternatively, whether a cell-intrinsic program is conferred onto the neuron from its precursor. To investigate these possibilities, we performed retroviral fate mapping and reconstructions of GCs generated in postnatal life in different regions of the SVZ. Our results indicate that at least two separate populations of precursors exist in the SVZ and give rise to GCs that target either the deep or the superficial lamina. Therefore, distinct precursors can produce one type of neuron, as defined by its morphology and intrinsic electric properties, but exhibiting different dendritic targeting. We performed heterochronic and heterotopic transplantations of different SVZ regions and observed that the majority of new GCs maintained their fate of targeting a specific lamina even when their precursors were grafted into SVZ environment that normally generated GCs with dendritic targeting of the opposite principal neuron lamina. Our results demonstrate that, in the mammalian brain, the pattern of dendritic targeting of a given neuron can be a cell-intrinsic property determined at the time of its birth. These findings have important implications, both for understanding the assembly of brain circuits during development and for the potential uses of adult neuronal stem cells in cell replacement therapies.

### One Type of Neuron with Two Different Patterns of Dendritic Targeting

Our findings indicate that the connectivity of one type of neuron as defined by its morphology and intrinsic somatic electrical properties in the same brain region, the GCs of the olfactory bulb, can in fact be determined by the particular population of neuronal precursor from which they derive. This observation suggests that neuronal precursors in the mammalian brain may be committed to produce neurons that are tailored to perform specific functions in a given neuronal microcircuit from as early as the time of their birth.

Our finding that lamina-specific dendritic targeting can be an intrinsic property of an immature neuron determined by the identity of its precursor has important implications for the logic of neuronal circuit assembly. In particular, GCs in the olfactory bulb that target the superficial lamina of the EPL are believed to establish synapses with tufted cells [16,34], whereas GCs that target the deep lamina are connected to mitral cells, and these two microcircuits are believed to serve different functions. The tufted-GC circuit is thought to be an intrabulbar association microcircuit [34] that may be important for low-threshold perception of odorants [35]. In contrast, the mitral-GC circuit is thought to mediate lateral inhibition and to participate in odor discrimination [36]. Thus, GCs that participate in specific microcircuits may be committed to their function already at the time of their birth from distinct populations of neuronal precursors.

To validate whether the neurons we labeled indeed constitute the same neuronal type with different dendritic targeting as suggested by their similar morphology, we measured their intrinsic somatic electrical properties, a useful feature for classification of neurons [26–28]. Indeed, labeled neurons with either deep or superficial dendritic targeting had similar intrinsic somatic electrical properties. This observation further suggests that one neuronal type (GC in the olfactory bulb) can exhibit two alternative patterns of dendritic targeting. This observation does not, however, preclude that minor differences may exist between GCs with deep and superficial dendritic targeting, such as differential expression of neurotransmitter receptor subunits [37]. In summary, our results suggest that distinct precursors can generate “tailor-made” neurons with different dendritic targeting connected to specific microcircuits. In addition, within one neuronal type, cells with alternative patterns of dendritic targeting may have subtle functional differences, such as differential expression of neurotransmitter receptors or ion channels, specific for their function in separate microcircuits.

### Mechanisms That Determine Dendritic Connectivity

Our findings also provide important insights into the developmental processes by which dendritic patterning is established in the mammalian brain. Particularly in comparison to axonal targeting, the mechanisms that regulate dendritic connectivity and allow neurons to establish proper contacts with their synaptic partners are not well understood [3]. Existing models for dendritic targeting can be divided into two major camps: outgrowth followed by pruning, or directed growth. For instance, in the mammalian retina, the dendritic arbor of retinal ganglion cells initially ramifies broadly, but as development proceeds, part of the dendritic branches are eliminated, such that the dendrites are ultimately segregated into two different laminae [7]. Additionally, a large body of work suggests that the refinement and stabilization of dendritic arbors may also be dependent on experience, a mechanism that would allow the maturing brain to adapt to a changing environment in postnatal life [4,10,11]. In other cases, the growth of dendrites can be targeted to specific laminae or layers in a directed manner. This mode of directed dendritic targeting has recently been demonstrated in the *Drosophila* olfactory system [38] and in the vertebrate retina [9]. For instance, in vivo imaging of zebrafish retinal development revealed that the dendrites of distinct classes of neurons directly grow to and innervate a specific lamina during their development [9].

How are such programs of dendritic development specified and implemented? Our experiments indicate that, in the mammalian olfactory bulb, the lamina-specific dendritic targeting of GC neurons is an intrinsic property determined by the precursor from which it arises. Our findings are compatible with both modes of dendrite growth described above. In one scenario, new neurons may directly extend their dendrites into the specific EPL lamina where they will form synaptic contacts. Alternatively, dendrites may initially grow in an exuberant manner through both laminae, but they will only form contacts with one type of principal neuron in either lamina, as determined by their precursors, and prune the rest of their dendritic arbors. Finally, after these lamina-specific dendritic contacts have been established, neuronal activity-dependent mechanisms then may play a role in the fine sculpting of GC dendritic arborization.

### Fate Restriction of Neuronal Precursors

The mechanisms that regulate the generation of neuronal diversity in the vertebrate nervous system have been investigated extensively. Various studies have shown that both the spatial and temporal origins of precursors determine the neurotransmitter phenotype, firing properties, calcium binding protein expression, and position of the cell body in different layers [28,39–43]. In particular for interneurons, distinct precursors defined by their expression of transcription factors give rise to specific types of interneurons for different brain regions [42]. Such specialization of precursors to produce different cell types persists throughout life in the SVZ for periglomerular and GC neurons [44–47]. While this work was under review, a study was published [48] demonstrating that the SVZ of postnatal animals has a mosaic organization, with different zones containing precursors committed to generate different types of periglomerular and granule neurons, as revealed by the presence of a set of immunocytochemical markers and the position of their cell bodies in the olfactory bulb. Our study advances previous observations [44,48] by demonstrating that the location of the dendrites and synapses of granule neurons is an intrinsic property of the cell, and by showing that there exist distinct precursors committed to generate neurons with dendritic targeting to specific laminae. Furthermore, we demonstrate that dendritic targeting is determined in the precursor cells in the lateral ventricle, before the progeny from these precursor cells have reached their target in the olfactory bulb. The hypothesis of the protomap, as originally proposed for the developing cortex, stated that the progenitors in the brain ventricles already contain the information [49,50] that specifies the identity of the neuronal cell types of the progeny that they will give rise to, their final destination in the different cortical layers, and the features specific to the different functional areas of the cortex. Our observations extend the protomap hypothesis by showing that the pattern of dendrite arborization can also be a feature already determined in the brain ventricles, before the progeny of the neuronal stem cells have reached their target. Furthermore, heterochronic as well as heterotopic transplantations of precursors confirmed that the fate of the dendritic targeting of a new neuron was maintained for the large majority of donor-derived GCs independent of the host environment in which their precursors had been grafted. These observations suggest that the connectivity of a neuron can be a cell-autonomous characteristic, determined by an intrinsic program in neonatal and adult neuronal stem cells.

Interestingly, we observed a small population of new GCs (<17%) derived from the neonatal aSVZ that displayed dendritic targeting to the opposite lamina both after heterochronic (into adult aSVZ) and heterotopic (into neonatal pSVZ) transplantation. In contrast, GCs derived after heterochronic and heterotopic transplantation of neonatal pSVZ and adult aSVZ donor tissue did not change their fate of dendritic targeting when challenged with a new SVZ environment. Several reasons could account for the small change of dendritic targeting after heterochronic or heterotopic transplantation from neonatal aSVZ donor tissue. First, a small population of aSVZ precursors could be reprogrammed to generate GCs with deep dendritic targeting after transplantation. Second, cells from the pSVZ, which migrate through the aSVZ, may be induced to proliferate and expand when transplanted, and this phenomenon could increase the number of GCs with deep dendritic targeting after transplanting the aSVZ. Third, a previously quiescent stem cell present in the neonatal aSVZ may be activated when exposed to a SVZ environment that generates GCs with deep dendritic targeting. Our experiments cannot currently distinguish between these and other possibilities, since the aSVZ tissue that is transplanted contains neuronal precursors at different stages of commitment, including rarely dividing stem cells, transient amplifying cells, and immature migrating neurons. In addition, by transplanting explants of tissues into a new SVZ environment, it is possible that donor cells surrounding the neuronal precursors could preserve the status of the donor niche, thus preventing the full reprogramming of the grafted progenitors. Nevertheless, our findings indicate that the precursors in the SVZ are committed to generate GCs with a prespecified pattern of dendrite targeting before they reach their target.

In recent years, there has been a surge in interest in the possibility of using different types of stem cells for cell replacement therapies aimed at correcting neurological disorders caused by neuronal loss, such as stroke and Parkinson, Huntington and Alzheimer diseases [51,52]. Our observations indicate that distinct neuronal stem cells are committed to generate not only a single neuronal type, but also cells with a prespecified pattern of dendritic targeting. Understanding the program by which neuronal stem cells specify how a neuron will target its dendrites towards a given synaptic partner could help to achieve neuronal replacement with cell type–specific connectivity. Further, the potential uses of endogenous adult neuronal stem cells/precursors for neuronal repair could be hindered by their lack of phenotypic plasticity as revealed by this and other recent studies [44–47]. Thus, the determination of cell type–specific dendritic connectivity by separate neuronal precursors may have important implications, both for the potential uses of adult neuronal stem cells in cell replacement therapies and for understanding the assembly of brain circuits during development.

## Materials and Methods

### Generation of retroviral vectors.

We used an oncoretroviral vector derived from the Moloney sarcoma virus expressing GFP under the control of the Rous sarcoma virus promoter (MolRG). Recombinant virus was prepared and stored as described [33]. The viral titer was 10^6^–10^7^ infectious units/μl.

### Stereotactic injections.

Animal care and procedures were approved by the local animal welfare committee. Neonatal (P5) and adult (>P56) Sprague-Dawley, Wistar Kyoto, and Lewis rats of either sex were anesthetized by hypothermia (neonatal rats) and with ketamine/xylazine (adult rats). In initial experiments, we injected P3 to P8 animals in the aSVZ and pSVZ. Between P3 to P8, we observed a superficial and deep branching population of GCs for aSVZ and pSVZ, respectively. For consistency, further experiments were performed at P5 for neonatal rats. Stereotactic injections were performed with a glass capillary with a tip diameter of 3–5 μm, and a volume of 0.1–0.5 μl of viral vector stock was injected. The following stereotactic coordinates (relative to bregma in millimeters) were used for neonatal animals: aSVZ: anterior 0.9, lateral ±2.1, ventral 2.1; pSVZ: posterior 0.6, lateral ±2.7, ventral 2.6; and for adult rats: aSVZ: anterior 1.2, lateral ±1.6 ventral 3.1; aRMS: anterior 2.8 lateral ±1.1, ventral 5.4; pRMS anterior 2.3, lateral ±1.4, ventral 4.5; pSVZ: posterior 2.7, lateral ±4.5, ventral 3.4. For neonatal sites of viral infection, see also Figure S1D. After surgery, animals were monitored for 24 h. Quantification of morphology of GCs was only performed at 14 d.p.i. and later time points in order to avoid including immature neurons that could still be migrating or had not yet acquired a mature morphology. After 14 d.p.i., most GCs from different origins (adult or newborn, aSVZ or pSVZ) had acquired a mature neuronal morphology (see Results). Infecting dividing precursors in the RMS within the adult olfactory bulb did not give rise to GFP^+^ GCs (*n* = 6 hemispheres injected; unpublished data), most likely due to the low level of proliferation in his region [20,53].

### Transplantation experiments.

FUGW^+^ transgenic rats [33] were bred on a Sprague-Dawley background. The aSVZ or pSVZ of neonatal and adult GFP^+^ rats was dissected (same regions as in Figure S1D), cut in small pieces, and then stereotactically transplanted into the aSVZ or pSVZ of either neonatal or adult wild-type Sprague-Dawley rats with the stereotactic coordinates described above.

### Tissue preparation and immunohistochemistry.

Rats were killed with ketamine/xylazine at the indicated time points for retroviral fate mapping or 35 d post-transplantation and perfused intracardially with 3% paraformaldehyde. After 24 h post-fixation in 3% paraformaldehyde at 4 °C, brains were cut into 50-μm coronal sections on a vibratome. Tissue sections were incubated with rabbit polyclonal anti-GFP antibody (1:3,000; Chemicon) in blocking solution containing phosphate buffered saline (PBS), bovine serum albumin, and 0.3% TritonX100 overnight at 4 °C, washed several times with PBS, and incubated with secondary anti-rabbit Alexa488 or Alexa555 conjugated antibody (1:750; Molecular Probes) for 2 h at room temperature. Tissue sections were washed in PBS and counterstained with Hoescht 33258 (Molecular Probes).

### Light microscopy, stereology, and neuronal reconstructions.

For stereological analysis and neuronal reconstructions, we used a Neurolucida system coupled to an inverted Olympus fluorescent microscope with a motorized X-Y-Z stage. For stereological analysis of the position of the soma and the initial branching point of the apical dendrite, we first determined the position of the soma and then traced the apical dendrite to its first branching point. All neurons of a tissue section that were not truncated before their initial dendritic branching were counted. For each time point, 250 neurons were traced from nine to 26 different olfactory bulb sections (*n* = 250 neurons for each time point from four to eight injected hemispheres from more than three animals) depending on the density of GFP^+^ GCs. For transplantation experiments, 200–731 neurons were traced. In initial experiments, we determined the distribution of the soma and the initial branching of the apical dendrite in serial sections throughout the anterior-posterior axes of the bulb. As we did not find any regional differences for the position of the soma and of the initial dendritic branching (unpublished data), we used sections from the central parts of the bulb for further analysis because they gave the highest yield of GFP^+^ GCs. In addition, we did not observe any differences in the soma distribution and the position of the initial dendritic branching point for animals of either sex, therefore data from both sexes were pooled. Host hemispheres differed in their density of GFP^+^ GCs. The distribution of deep and superficial GCs did not, however, differ regardless of the cell density in the same transplantation condition. For each tissue section, we traced the borders of the different layers based on the nuclear counterstaining with Hoechst 33258 (see also Figure 2). Based on these borders, we divided the granule cell layer (GCL) in percentages: 0% being the border between the RMS and GCL, and 100% being the MCL. For our analysis, dividing the internal plexiform layer (IPL) and the GCL did not prove useful because many of the GC somata were located in the level of the IPL or around the MCL. Based on these borders, we divided the EPL in percentages: 0% being the MCL and 100% being the border between the EPL and the GL. For further analysis, the GCL and the EPL were divided in 10% steps, and the position of the somata and of the initial branching of the apical dendrite was plotted as a cumulative distribution. We calculated the differences in percentages of the ratio of GCs that initially branched below 50% (40%) of EPL. The lower threshold (40%) of EPL gave similar results (±3.4%) to the 50% threshold. GCs for single-cell reconstructions were selected based on the typical position of the soma and on the initial branching of the apical dendrite as found for the respective population. Ten GCs for each time point or transplantation condition without apparent truncation due to tissue sectioning were then reconstructed. Of these GCs, we marked spine protrusions manually with a 40× lens while continuously adapting the *z*-axis. All spine-like protrusions were counted that emerged from the dendrites and had a thickness and morphology that would make them appear as spine- or filapodia-like structures. The distribution of spines of the apical dendrite for each GC was attributed to percentage ranges in the EPL (as defined above, here 20% steps) and then the distribution of spines in the EPL for ten GCs was averaged for each time point or transplantation condition. Statistical significance (*p* < 0.05) was determined with a nonparametric Mann-Whitney test for unpaired samples.

### Electrophysiological recordings.

Rat pups were bilaterally injected with 1 μl of oncoretroviral vector expressing GFP in aSVZ and pSVZ in neonatal rats. At 21 to 23 d.p.i., animals were anesthetized with isofluorane, and brains were rapidly removed. The 350-μm horizontal olfactory bulb slices were cut with a Leica vibratome in cutting solution containing (in mM): 212 sucrose, 3 KCl, 1.25 NaH_2_PO_4_, 26 NaHCO_3_, 7 MgCl_2_, 0.5 CaCl_2_, 10 glucose, 310 mOsm, (pH 7.3). Slices were recovered for 30 min at 32 °C with recording solution containing (in mM): 125 NaCl, 2.5 KCl, 1.25 NaH_2_PO_4_, 26 NaHCO_3_, 1 MgCl_2_, 2 CaCl_2_, 20 glucose, 310 mOsm, (pH 7.3) and continuously bubbled with carbogen. After recovery, slices were kept at room temperature.

Targeted whole-cell recordings were performed on GFP^+^ nontruncated CGs with a MultiClamp700B amlpifier (Axon Instruments) and pipette solution containing (in mM): 2 NaCl, 4 KCI, 130 K-gluconate, 10 HEPES, 0.2 EGTA, 4 ATP-Mg, 0.3 GFP-Tris, 14 phosphocreatine, 0.02Alexa555 hydrazide, 292 mOsm, and pH 7.25. Pipette resistance was 6-9 MW. Access resistance was 12-30 MW, which was not compensated and regularly monitored during recordings. Liquid junction potential was not corrected. Data were acquired and analyzed with the pClamp9 software (Axon Instruments). Neurons were considered to hav *I* current if in a voltage ramp (10 mV steps for 400ms), the peak-to-plateau ratio was > 2. After recordings, the tissue was fixed and the neurons were reconstructed. Recordings from the correct GCs were conffirmed by coocalization of Alexa555 flourescence with GFP^+^ GCS.

## Supporting Information

Figure S1 Distinct Precursors Gave Rise to GCs with Initial Branching of the Apical Dendrite (1.2 MB TIF)

Figure S2 Distribution of Postsynaptic Sites in the Dendrites of GCs. (4.6 MB TIF)

Text S1 Lamina-Specific Distribution of Synapses. (120 KB DOC)

## Abbreviations

aSVZ: anterior subventricular zone
d.p.i.: days postinfection
EPL: external plexiform layer
GC: granule cell
GCL: granule cell layer
GFP: green fluorescent protein
MCL: mitral cell layer
P: postnatal day
pSVZ: posterior subventricular zone
RMS: rostral migratory stream
SVZ: subventricular zone
