# Correction: Distinct Mammalian Precursors Are Committed to Generate Neurons with Defined Dendritic Projection Patterns

**DOI:** 10.1371/journal.pbio.0050336

**Published:** 2007-12-27

**Authors:** Wolfgang Kelsch, Colleen P Mosley, Chia-Wei Lin, Carlos Lois

Correction for:

Kelsch W, Mosley CP, Lin CW, Lois C (2007) Distinct Mammalian Precursors Are Committed to Generate Neurons with Defined Dendritic Projection Patterns . PLoS Biol 5(11): e300. doi:10.1371/journal.pbio.0050300

There was an error in the last line of the legend for Figure 4C. It should read “…ISI, the interspike interval in milliseconds…”

There were also several errors in the final paragraph of the Materials and Methods section. Lines 6 and 7 should read:

“Pipette resistance was 6–9 MΩ. Access resistance was 12–30 MΩ, not compensated and regularly monitored during recordings.”

