# Correction: Distinct Mammalian Precursors Are Committed to Generate Neurons with Defined Dendritic Projection Patterns

**DOI:** 10.1371/journal.pbio.0060091

**Published:** 2008-04-29

**Authors:** Wolfgang Kelsch, Colleen P Mosley, Chia-Wei Lin, Carlos Lois

## Abstract

.

Correction for:

Kelsch W, Mosley CP, Lin CW, Lois C (2007) Distinct mammalian precursors are committed to generate neurons with defined dendritic projection patterns. PLoS Biol 5(11): e300. doi:10.1371/journal.pbio.0050300

## 

**Figure 1:**
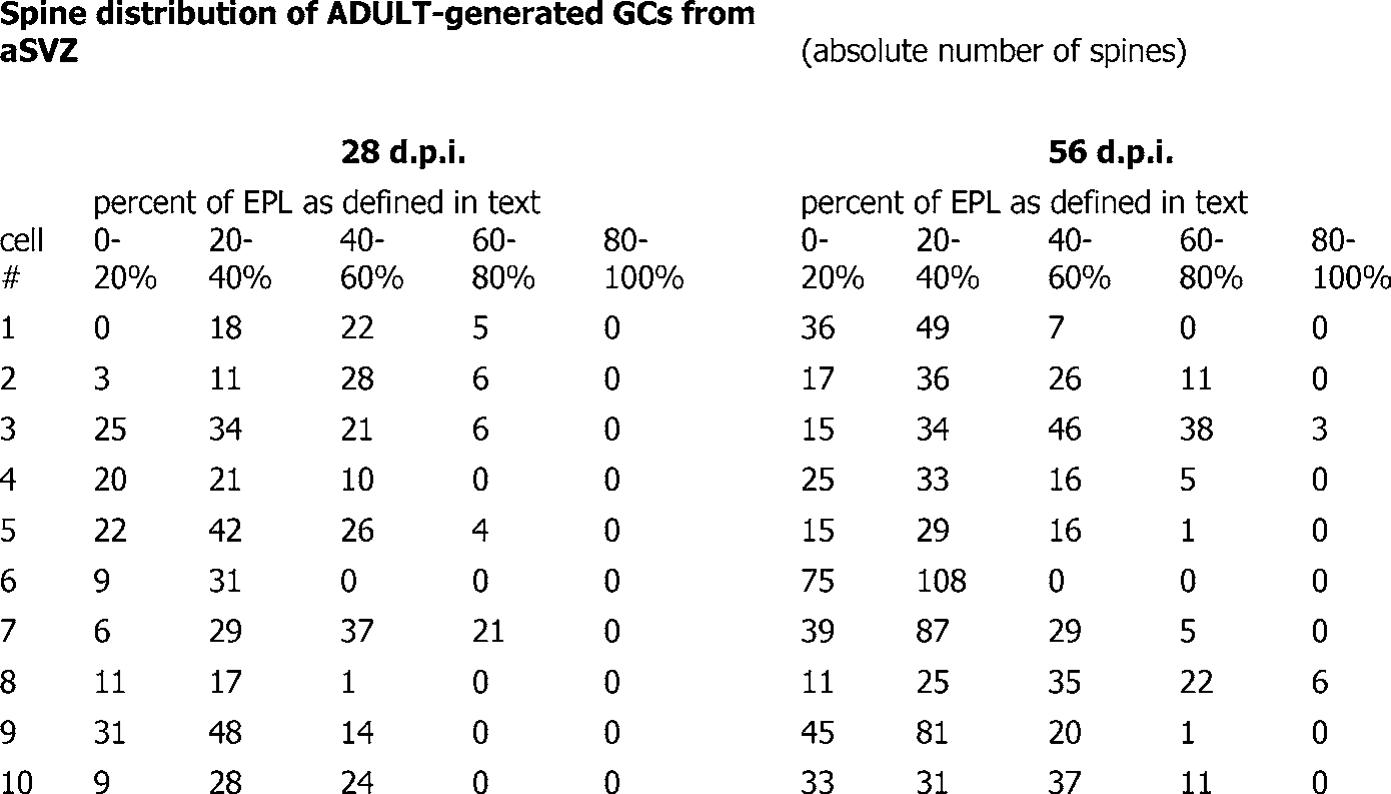
Raw Data During the preparation of the graphs, the wrong dataset was plotted in the right-hand graph of Figure 3 for the spine distribution of different GC populations. The correct raw data are shown.

**Figure 2:**
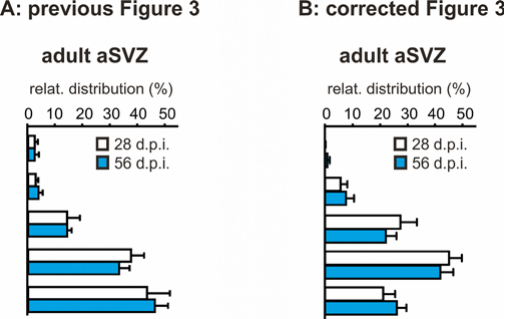
Revised Figure 3 The previously published graph found on the far right of Figure 3 (A) and the corrected graph (B) for the spine distribution of different adult-generated GC populations (for 28 and 56 days post infection (d.p.i.) are shown. We regret any confusion this may have caused.

