# Supplementary material for: Distinct Mammalian Precursors Are Committed to Generate Neurons with Defined Dendritic Projection Patterns
Source: PLoS Biol. 2007 Nov 13;5(11):e300. doi: 10.1371/journal.pbio.0050300 (PMC2071944; doi:10.1371/journal.pbio.0050300)
Supplement: Supplementary file 1 [file pbio.0050300.sd001.doc]

**Supplemental Text S1**

**Distribution of postsynaptic densities of glutamatergic input synapses in the EPL.**

To test whether spine distribution reflects the distribution of dendro-dendritic synapses, we genetically labeled GCs with the fusion protein PSD-95:GFP. PSD-95 is a protein enriched in the postsynaptic density of glutamatergic synapses, and thus PSD-95:GFP has been used as a genetic marker to identify glutamatergic postsynaptic sites [1-3]. In the dendro-dendritic synapses of the GCs, the lateral dendrites of the bulb’s projection neurons (mitral and tufted cells) provide glutamatergic input to the GCs, and, in turn, the GCs release GABA back onto the projection cells. PSD-95:GFP^+^ clusters were found in the superficial lamina of the EPL for GCs that originated from precursors infected in aSVZ at P5 (Figure S2B), whereas PSD-95:GFP^+^ clusters were found in the deep lamina of the EPL for GCs from precursors infected in adult aSVZ (Figure S2C).

**Supplemental Experimental Procedures**

***Generation of lentiviral vectors****.* cDNA of PSD-95:GFP [4]. The cDNA of PSD-95:GFP was amplified by PCR. Then, the GFP sequence of the MolRG was replaced by PSD-95:GFP. The resulting construct is called MolRPSD-95:GW.

***Confocal imaging.*** Confocal image stacks were acquired using an Olympus Fluoview confocal microscope (Olympus, Melville, NY) from processed tissue as described. A typical image stack consisted of 60 image planes each of 1024x1024 pixels (pixel size, 0.23x 0.23 µm) with z-step 0.25 µm. For each stack, laser intensity and detector sensitivity were set to values of comparable background intensity. PSD-95:GFP was previously used to label postsynaptic densities in vivo [1,2]. We confirmed that PSD-95:GFP expression by lentiviral vectors in vitro did not change the average AMPA receptor mediated mEPSC amplitude and frequency in cultured neurons PSD-95:GFP clusters contacted a presynaptic marker, bassoon, reliably and in vivo (W. Kelsch, C. Lois, manuscript in preparation). In addition, visualizing the dendritic arbor by staining the diffuse PSD-95:GFP protein in the dendrites with red immunofluorescence against GFP did not interfere with the fluorescence of PSD-95:GFP^+^ clusters (Figure S2A).

**Supplemental Figure Legends**

**Figure S1	Distinct precursors gave rise to GCs with initial branching of the apical dendrite.**

**A**	Left: Cumulative distribution of initial branching point of the dendrite in the EPL of GCs generated in adult (blue) and P5 (red) *Wistar Kyoto* and *Lewis* inbred rat strains. Right: Cumulative distribution of the soma position in the GCL of GCs generated in adult (blue) and P5 (red) inbred rat strains shown in A.

**B**	Left: Cumulative distribution of initial branching point of the dendrite in the EPL of GCs generated in adult anterior and posterior RMS (aRMS and pRMS, respectively) and pSVZ (21 d.p.i.). Right: Cumulative distribution of the soma position in the GCL of GCs generated in adult aRMS, pRMS and pSVZ.

**C**	Reconstructions of newly generated GCs from the aRMS (21 d.p.i.) with

deep and superifical (asterisk) dendritic targeting of the EPL (bar = 100 µm).

**D**	Sites of viral infection in the neonatal anterior and posterior SVZ.

**Figure S2	Distribution of postsynaptic sites in the dendrites of GCs.**

**A**	Postsynaptic densities of glutamatergic synapses where genetically labeled by infecting GC precursors in the SVZ with a retrovirus encoding a PSD-95:GFP (28 d.p.i). PSD-95:GFP^+^ fluorescence occurred in clusters (green) at the tip of the spines. To reveal the dendritic arbor we took advantage of the fact that we could visualize the dendritic cytoplasmic PSD-95:GFP protein using immunofluorescence with an antibody raised against GFP (red). The confocal pictures were then merged and revealed postsynaptic densities of glutamatergic input synapses in green (green intrinsic fluorescence of PSD-95:GFP) and yellow (green fluorescence of PSD-95:GFP plus red signal of the immunofluorescence) and the dendritic arbor in red (immunofluorescence) (bar = 10 µm).

**B**	Postsynaptic densities of glutamatergic synaptic input were confined to the superficial lamina of EPL for GCs neurons with superficial initial dendritic branching from P5 aSVZ. Some of the distal dendrites reached the lower region of the glomeruli (bar = 20 µm).

**C**	Postsynaptic densities in the deep lamina of the EPL for GCs with initial branching below or around the mitral cell layer as typically found for GCs from adult aSVZ precursors (bar = 20 µm).

**Supplemental Material References**

1	Niell CM, Meyer MP, Smith SJ (2004) In vivo imaging of synapse formation on a growing dendritic arbor. Nat Neurosci 7(3): 254-260.

2	Gray NW, Weimer RM, Bureau I, Svoboda K (2006) Rapid redistribution of synaptic PSD-95 in the neocortex in vivo. PLoS Biol 4(11): e370.

3	Okabe S, Kim HD, Miwa A, Kuriu T, Okado H (1999) Continual remodeling of postsynaptic density and its regulation by synaptic activity. Nat Neurosci 2(9): 804-811.

4	Hsueh YP, Sheng M (1999) Requirement of N-terminal cysteines of PSD-95 for PSD-95 multimerization and ternary complex formation, but not for binding to potassium channel Kv1.4. J Biol Chem 274(1): 532-536.
